# Sex Hormones Enhance Gingival Inflammation without Affecting IL-1*β* and TNF-*α* in Periodontally Healthy Women during Pregnancy

**DOI:** 10.1155/2016/4897890

**Published:** 2016-03-01

**Authors:** Min Wu, Shao-Wu Chen, Wei-Lan Su, Hong-Ying Zhu, Shu-Yuan Ouyang, Ya-Ting Cao, Shao-Yun Jiang

**Affiliations:** ^1^Department of Stomatology, The Affiliated Shenzhen Maternity and Child Healthcare Hospital of the South Medical University, Shenzhen 518048, China; ^2^Department of Obstetrics and Gynecology, The Affiliated Shenzhen Maternity and Child Healthcare Hospital of the South Medical University, Shenzhen 518048, China; ^3^Clinical Laboratory, The Affiliated Shenzhen Maternity and Child Healthcare Hospital of the South Medical University, Shenzhen 518048, China; ^4^Hospital of Stomatology, School of Dentistry, Tianjin Medical University, Tianjin 300070, China

## Abstract

Hormones (progesterone and estradiol) change greatly during pregnancy; however, the mechanism of hormonal changes on gingival inflammation is still unclear. This study is to evaluate the effects of hormonal changes during pregnancy on gingival inflammation and interleukin-1*β* (IL-1*β*) and tumor necrosis factor-*α* (TNF-*α*) in gingival crevicular fluid (GCF). 30 periodontally healthy pregnant women were evaluated in the first, second, and third trimesters. 20 periodontally healthy nonpregnant women were evaluated twice (once per subsequent month). Clinical parameters including probing pocket depth (PPD), bleeding index (BI), gingival index (GI), clinical attachment level (CAL), and plaque index (PLI) were recorded. GCF levels of IL-1*β* and TNF-*α* and serum levels of progesterone and estradiol were measured. From the data, despite low PLI, BI and GI increased significantly during pregnancy; however, no significant changes in PLI, CAL, IL-1*β*, or TNF-*α* GCF levels were observed. Although IL-1*β*, not TNF-*α*, was higher in pregnant group than in nonpregnant group, they showed no correlation with serum hormone levels during pregnancy. GI and BI showed significant positive correlation with serum hormone levels during pregnancy. This study suggests that sex hormone increase during pregnancy might have an effect on inflammatory status of gingiva, independent of IL-1*β* and TNF-*α* in GCF.

## 1. Introduction

Since the 1960s, it has been proposed that periodontal health is associated with pregnancy [1, 2]. It is widely accepted that preexisting gingivitis or periodontitis in women would be worsening dramatically during pregnancy. Taani et al. have summarized that the prevalence of gingivitis during pregnancy ranged widely from 35 to 100% [3]. Though the exact mechanisms of exacerbating gingival inflammation during pregnancy have not yet been completely elucidated, it was supposed in the 1970s that the increase in serum estrogen and progesterone had a dramatic effect on the periodontium throughout pregnancy, which was correlated with clinical signs [4, 5]. However, some studies demonstrated no obvious gingival changes during pregnancy compared with nonpregnant controls [6, 7]. Thus, the correlation between hormone levels during pregnancy and gingival inflammation remains controversial.

Investigators have reported that increased female sex hormones may modulate the function of immune cells [8, 9]. Immunological changes during pregnancy have been considered to be, at least in part, responsible for periodontal conditions [10]. Meanwhile, proinflammatory cytokines play a major role in the progression of gingival inflammation [11]. Interleukin- (IL-) 1*β* and tumor necrosis factor- (TNF-) *α* regulate the initial stages of inflammation by increasing the recruitment of neutrophils and monocytic phagocyte [12].

The effects of hormones on these cytokines in periodontium have been studied extensively in vitro. Morishita et al. reported that estradiol at 0.04 ng/mL or more inhibited IL-1 secretion, and progesterone at 0.1 ng/mL or more and 0.02 ng/mL or more, respectively, suppressed the production of IL-1*α* and IL-1*β* induced by lipopolysaccharides (LPS) in human monocytes [13], which indicates that high levels of estradiol and progesterone inhibited IL-1 secretion in human peripheral monocytes stimulated by LPS. In vitro study showed that sex hormones at physiological concentrations (estradiol of 10^−9^ to 10^−7 ^M) had an inhibitory effect on the secretion of IL-1*β* and TNF-*α* by human periodontal ligament cells treated with* E. coli* LPS [14]. Also, Smith et al. found that TNF-*α* level in blood neutrophils decreased when estrogen and progesterone concentration were elevated [15]. These in vitro studies mentioned above focused on the effect of sexual hormones on cytokines under the challenge of bacteria.

As for human studies, many researchers investigated the change of inflammatory cytokines in pregnant women with gingivitis or periodontitis. A significant impact of periodontal therapy such as scaling and planning on the levels of IL-1*β* in gingival crevicular fluid was observed in pregnant women with periodontitis [16, 17]. Also, it is well known that gingival inflammation associated with pregnancy has been initiated by dental plaque and exacerbated by endogenous steroid hormones [18]. These studies did not exclude the effects of previously existing periodontal inflammation and dental plaque. It has been already reported that good oral hygiene in pregnancy was able to partially neutralize hormonal effect [19]. In early reports, some authors stated that healthy gingiva was not affected by pregnancy and the incidence of gingivitis was only 0.03% if a plaque-free state was maintained [4, 20]. Nevertheless, the sole effect of sex hormones on gingival inflammation is still unclear. Meanwhile, the research evaluating the change of periodontal status and local inflammatory responses in periodontally healthy women during pregnant is scarce. Thus, in this study, we collected women with healthy periodontium and excellent oral hygiene, to evaluate the effect of hormonal changes occurring during pregnancy on gingival inflammation and GCF levels of IL-1*β* and TNF-*α*.

## 2. Material and Methods

### 2.1. Subjects

Ethical approval was obtained from the Research and Ethics Committee of Shenzhen Maternity and Child Healthcare Hospital (China, approval number: 19) in full accordance with the World Medical Association Declaration of Helsinki (version 2002). With informed consent, the volunteers with excellent oral hygiene, periodontal health, and no smoking were recruited from June 2010 to June 2012 in Shenzhen Maternity and Child Healthcare Hospital. Exclusion criteria were systemic or topical antimicrobial/anti-inflammatory therapy within the previous 3 months, chronic systemic disease (e.g., diabetes, hypertension, epilepsy, cardiac disease, lung disease, and renal disease), and positive test for human immunodeficiency virus (HIV), multifetal gestation.

During the observation period, subjects who had average PI scores >1 were excluded from the study. In pregnant group (Pr group), thirty pregnant women (aged 25 to 35) with gestational age at 12–14 weeks were recruited. Gestational age was determined according to information of sequential physical exams, data from menstrual cycles, and ultrasound test [18]. In nonpregnant control group (N-Pr group), 20 volunteers were selected in the same dental department. There was no difference in social-economical situation between two groups (Table 1).

The women in Pr group were examined three times during pregnancy (Pr I: 12–14 weeks; Pr II: 23–25 weeks; and Pr III: 33–36 weeks (gestational age)) [21]. The women in N-Pr group were examined twice (N-Pr I and N-Pr II) around the luteal period of the menstrual cycle, once per subsequent month [22]. At each examination, serum and gingival crevicular fluid (GCF) samples and clinical data were collected. Meantime, oral hygiene instructions were performed.

### 2.2. Clinical Measurements

Periodontal examination was performed by the same periodontists, using a manual periodontal probe (Kangqiao, Shanghai, China). The following clinical parameters were recorded at three sites (mesiobuccal, buccal, and distobuccal) of each tooth, excluding the third molar: plaque index (PLI): “0” = an absence of plaque on the clinical crown, “1” = the presence of soft deposits covering gingival margin, “2” = the presence of soft deposits covering between one-third and two-thirds of the crown, and “3” = the presence of soft deposits covering more than two-thirds of the crown [23]; gingival index (GI): “0” = normal gingival, “1” = mild inflammation, “2” = moderate inflammation, and “3” = severe inflammation [23]; bleeding index (BI): “0” = no bleeding, “1” = the presence of bleeding as a single point, “2” = the presence of bleeding as a thin line, and “3” = the presence of profuse bleeding as an immediate flow [22]; probing pocket depth (PPD): defined as the distance from the free gingival margin to the bottom of the sulcus; clinical attachment level (CAL): defined as the distance from the cemento-enamel junction to the bottom of the sulcus.

### 2.3. GCF Sampling

At each visit, GCF samples were collected from the mesiobuccal sites of upper premolars (14, 15, 24, and 25) before clinical measurements, using Waterman III filter-paper (Whatman International Ltd., Maidstone, England) (four samples per patient and per visit). Prior to collecting GCF, the sampling sites were gently air-dried. One filter-paper strip per each sampling site was used. The strip was inserted into gingival sulcus until slight resistance was felt and left there for 30 seconds. The samples contaminated with blood were discarded and a substituted GCF sample was taken from the mesiobuccal sulcus of the adjacent upper canine (13 or 23). The strips of each subject were immediately placed into two sterilized Eppendorf tubes and kept at −70°C.

### 2.4. Serum Sampling

Blood samples were collected in the morning at the same examination. 5 mL of blood was drawn from every subject into a free-anticoagulant vacuum tube. Serum was obtained after immediate centrifugation at 3000 g for 5 min and stored at −70°C until further evaluation.

### 2.5. IL-1*β* and TNF-*α* Assessment

GCF samples were extracted from the paper strips by eluting with 200 *μ*L of phosphate-buffered saline and 2 *μ*L of phenylmethanesulfonyl fluoride (20 mM) and incubated for 30 min at 4°C. Then, the tubes were centrifuged at 19,000 g for 5 min. The supernatants were collected and used to measure IL-1*β* and TNF-*α* level. The concentrations of IL-1*β* and TNF-*α* level were determined by using commercially enzyme-linked immunosorbent assay (ELISA) kits (R&D, MN, USA) according to the protocol.

### 2.6. Estradiol and Progesterone Assays

After serum samples thawed at room temperature and centrifuged at 4000 g for 5 min, supernatants were extracted for measurement of estradiol and progesterone by means of chemiluminescence method (Beckman Coulter Inc., MN, USA).

### 2.7. Statistical Analyses

Data were presented as mean and standard deviations. *t*-test or Analysis of Variance (ANOVA) was used for data. Correlations among the clinical parameters, GCF cytokines, and serum hormones were evaluated with Pearson's test. *P* values < 0.05 were considered statistically significant.

## 3. Results

### 3.1. Periodontal Parameters

At the first visit, all subjects had 28–32 teeth and the periodontal examination was in Table 2, which showed that there were no differences in PLI, PPD, GI, BI, and CAL (CAL = 0). During the pregnancy, PLI did not change compared to N-Pr group (*F* = 0.64, *P* = 0.6373), which indicated that all subjects kept good hygiene (Table 3). Although PPD had the increasing tendency, the difference was not significant (*F* = 2.40, *P* = 0.0536) (Table 3). GI and BI increased significantly (*F* = 19.76, *P* < 0.05; *F* = 19.98, *P* < 0.001) during pregnancy, which was higher than in the N-Pr group (Table 3). No changes in CAL were detected during the follow-ups (CAL = 0).

### 3.2. Inflammatory Cytokines in GCF

Table 4 showed the concentrations of IL-1*β* and TNF-*α* in the Pr group and the N-Pr group. Compared with the N-Pr group, there were no significant changes in GCF TNF-*α* level in Pr group (*F* = 0.45, *P* = 0.7726); however, GCF IL-1*β* level increased obviously (*F* = 7.41, *P* < 0.0001). During pregnancy, the GCF IL-1*β* and TNF-*α* levels in three trimesters did not show significant difference.

### 3.3. Hormonal Levels in Serum and Correlation with Gingival Inflammation during Pregnancy

Serum estradiol and progesterone concentrations in the Pr group were higher than in the N-Pr group. Furthermore, serum estradiol and progesterone levels increased gradually during pregnancy (*F* = 73.87, *P* < 0.0001; *F* = 64.23, *P* < 0.0001) (Table 5).

In the Pr group, positive correlation was found between gingival inflammation (GI and BI) and serum estradiol level during pregnancy (*r* = 0.695, *P* < 0.0001; *r* = 0.683, *P* < 0.0001), while no obvious correlation was found between PPD and serum estradiol level (*r* = 0.23, *P* = 0.222). Positive correlation was found between gingival inflammation (GI and BI) and serum progesterone level during pregnancy (*r* = 0.694, *P* < 0.0001; *r* = 0.683, *P* < 0.0001), while no obvious correlation was found between PPD and serum progesterone level (*r* = 0.23, *P* = 0.222) (Table 5). Because no changes were observed in GCF IL-1*β* and TNF-*α* level during pregnancy, they could not be correlated with the increase in gingival inflammation or in serum hormones.

## 4. Discussion 

This study describes the changes in periodontal parameters and GCF inflammatory cytokines during pregnancy in periodontally healthy women and the correlation among serum hormonal levels, periodontal parameters, and inflammatory cytokines.

As some investigations showed, repetition and reinforcement of oral hygiene instructions were critical in improving oral hygiene and were able to reduce clinical signs of gingivitis in pregnant women and other nonpregnant subjects [24, 25]. In this study, participants persisted in plaque control and kept low plaque index, which eliminated the effects of bacteria on periodontal inflammation as much as possible. From our data, even though the PLI in pregnancy women was similar to in nonpregnancy women, pregnant women had elevated gingival inflammation, which further confirmed the hypothesis that pregnancy may be associated with inflammatory changes in gingival tissues. Also, our data were consistent with the previous researches [21, 22]. In these two researches, gingival inflammation during pregnancy was examined, in which healthy periodontium and good oral hygiene were included in the subject criteria. Figuero et al. [21] found that GI increased, despite of fairly low Pl values, and maintained high levels in the third trimester in 48 pregnant Spanish women. However, the data about PPD was not reported [21]. In the other longitudinal study, gingival inflammation in 30 periodontally healthy pregnant women with good oral hygiene in Finland was evaluated by bleeding on probing values and proportion of periodontal pockets (≥4 mm) which increased without relation to plaque between the first and second trimesters [22]. The data about PPD were not consistent with our data. In this present study, PPD showed an increasing tendency in these three trimesters; however, there was no significant difference among three stages. It is possibly due to the different race and different reaction to hormonal levels. Meanwhile, although in some previous cross-sectional and longitudinal studies on subjects with gingivitis and periodontitis, PPD significantly increased during pregnancy [1–4], it was explained that the PPD change was induced by bacteria and increased hormones simultaneously.

Pregnant women showed significantly increased gingival inflammation (GI, BI), which reached high levels in the third trimester with little change in attachment level, although plaque scores were nearly unchanged and kept fairly low (PLI < 1). The levels of GI and BI in early pregnancy were already higher than that of nonpregnant women. It suggested that the early pregnancy had already affected the gingiva. However, gingival pockets (>3 mm) and GI values (>2) were not found in the study, indicating that pregnancy has limited influence on gingival inflammation, when good plaque control was maintained. Additionally, no change in CAL was detected during the follow-ups, which was in agreement with most previous reports [3, 26]. Miyazaki et al. [7] suggested that the increase of periodontal pocket during pregnancy is caused by enlargement of gingival tissue rather than periodontal destruction, which was confirmed in our study. It could be speculated that a chronic and lasting inflammatory state of the gingiva is essential to cause attachment loss.

Since the localization of estrogen receptor and progesterone receptor has been reported in the human periodontium, Preshaw [27] considered that the obvious increase in circulating levels of estrogen and progesterone was supposed to have a dramatic effect on the periodontium throughout pregnancy and be correlated with clinical phenomenon. In our results, serum estradiol and progesterone levels increased greatly during the pregnancy as expected and were much higher than those of nonpregnant group. Positive correlation was found between the increased inflammation in gingiva and the increase in serum estradiol and progesterone levels during pregnancy. Sexual hormones levels could be the factors that are responsible for the increase of gingival inflammation. Vogt et al. [28] mentioned that sexual hormones would influence the inflammatory status of healthy gingiva in a limited degree when good plaque control was maintained, which is consistent with our study. GI and BI, not PPD, changed significantly during pregnancy, which might be explained by the impact of sexual hormones on vascular permeability [12]. However, in the aforementioned study from Spain, no significant correlation was found between GI increase and salivary hormone levels. It is possible that salivary hormone levels are different from serum hormone levels, resulting in different results.

Furthermore, we explored the changes of inflammatory cytokines in GCF during pregnancy. One longitudinal study, on 18 premenopausal women, observed that the GCF levels of IL-1*β* and TNF-*α* remained stable between ovulation and progesterone peak [11]. Thus, in our study, the nonpregnant women during the luteal period of the menstrual cycle according to Gürsoy et al. [22] represented the comparable women before gestation. In the present study, there were no remarkable differences in the GCF IL-1*β* levels among three trimesters in pregnant women, although their concentrations were significantly higher than found in nonpregnant women, which is in agreement with the results of Figuero et al. [21]. At the first visit, GI and BI had no significant difference between N-Pr and Pr group; however, GCF IL-1*β* level had increased in Pr group. Afterward, although GCF IL-1*β* level kept stable during pregnancy, its level was higher than in N-Pr group. As Boronat-Catalá et al. [29] mentioned that IL-1*β* in GCF could be used as a reliable marker of the degree of inflammation in gingivitis, we considered that increased IL-1*β* was involved in the gingival inflammation in pregnant women. Under the strict control of dental plaque during the observation period, an elevated level of GCF IL-1*β* in the first trimester suggested that the early pregnancy had already affected GCF IL-1*β*. However, whether the increase of GCF IL-1*β* was induced by sex hormone was unknown. Shu et al. [14] demonstrated that sex hormones at physiological concentrations (Estradiol of 10^−9^ to 10^−7 ^M) had an inhibitory effect on the secretion of IL-1*β* by human periodontal ligament (hPDL) cell. Meanwhile, from some studies, estrogen had no effect on IL-1*β* or negatively regulated its secretion [30, 31]. Thus, we inferred that IL-1*β* increase was not induced by sex hormone. The mechanism needs further to be explored. The reason for stable GCF IL-1*β* level during pregnancy could be explained as following. First, immunosuppression, to some extents, occurs in pregnancy [32] and suppresses the further increase in IL-1*β* level. Second, high levels of estradiol and progesterone inhibited further IL-1 secretion, which is consistent with previous research [13, 14].

TNF-*α* stimulates collagenase production and bone resorption and impairs the repair capacity of the periodontium. In samples from nonpregnant women, GCF TNF-*α* level could not be classified as a marker of inflammation in gingivitis [30]. GCF TNF-*α* level is more associated with alveolar bone resorption and attachment loss. In our study, GCF TNF-*α* level did not change during pregnancy, which could explain the reason that no attachment loss occurred. Furthermore, no positive correlation between serum hormones and GCF TNF-*α* level was found. It is possible that elevating estrogen and progesterone inhibited TNF-*α* secretion, which was described in blood neutrophils [14].

## 5. Conclusion

Sexual hormones estradiol and progesterone would influence the inflammatory status of gingiva even under good oral hygiene control during pregnancy, independent of GCF levels of IL-1*β* and TNF-*α*.

## Figures and Tables

**Table 1:** Social characteristics of the groups studied.

Social variable	Nonpregnant women	Pregnant women
Age (years)	29.250 ± 2.314	28.333 ± 1.971
Ethnicity	Han	Han
Occupation		
Housewife	0	3
Employee	20	27
Level of education		
<Technical	6	10
Bachelor	9	13
Master	5	7
Economic status	Regular	Regular

**Table 2:** Clinical periodontal parameters ($\bar{x}\pm s$) in the groups.

Variable	N-Pr group (*n* = 20)	Pr group (*n* = 30)	*t* value	*P*
PLI	0.570 ± 0.060	0.598 ± 0.098	0.67	0.5087
GI	1.250 ± 0.126	1.205 ± 0.109	1.33	0.1907
BI	1.145 ± 0.079	1.217 ± 0.135	−1.27	0.2092
PPD (mm)	2.235 ± 0.253	2.282 ± 0.267	0.79	0.4343
CAL (mm)	0	0	—	—

Pr group: pregnancy group; N-Pr group: nonpregnancy group; PLI: plaque index; GI: gingival index; BI: bleeding index; PPD: periodontal pocket depth; CAL: clinical attachment loss; *n*: number.

**Table 3:** Periodontal parameters in pregnant group and nonpregnant group ($\bar{x}\pm s$).

Group	PPD (mm)	GI	BI	PLI
Pr group (*n* = 30)				
First trimester	2.282 ± 0.267^a^	1.205 ± 0.109^a^	1.217 ± 0.135^a^	0.598 ± 0.098^a^
Second trimester	2.336 ± 0.250^a^	1.357 ± 0.152^b^	1.360 ± 0.194^b^	0.603 ± 0.110^a^
Third trimester	2.411 ± 0.249^a^	1.485 ± 0.169^c^	1.510 ± 0.223^c^	0.610 ± 0.097^a^
N-Pr group (*n* = 20)				
First month	2.235 ± 0.253^a^	1.250 ± 0.126^a^	1.145 ± 0.079^a^	0.570 ± 0.060^a^
Second month	2.225 ± 0.233^a^	1.235 ± 0.108^a^	1.237 ± 0.113^a^	0.590 ± 0.076^a^
*F*	2.40	19.76	19.98	0.64
*P*	0.0536	<0.0001	<0.0001	0.6373

Pr group: pregnancy group; N-Pr group: nonpregnancy group; PPD: periodontal pocket depth; GI: gingival index; BI: bleeding index; PLI: plaque index. Different letters represent significant difference.

**Table 4:** IL-1*β* and TNF-*α* level in pregnant group and nonpregnant group ($\bar{x}\pm s$).

Group	IL-1*β* (ng/L)	TNF-*α* (ng/L)
Pr group (*n* = 30)		
First trimester	11.192 ± 3.186^b^	167.111 ± 68.733^a^
Second trimester	11.033 ± 2.647^b^	158.124 ± 46.857^a^
Third trimester	11.368 ± 2.632^b^	173.455 ± 52.738^a^
N-Pr group (*n* = 20)		
First month	8.031 ± 3.509^a^	166.072 ± 39.098^a^
Second month	7.972 ± 3.758^a^	156.427 ± 51.091^a^
*F*	7.41	0.45
*P*	<0.0001	0.7726

Pr group: pregnancy group; N-Pr group: nonpregnancy group; IL-1*β*: interleukin-1*β*; TNF-*α*: tumor necrosis factor-*α*; Different letters represent significant difference.

**Table 5:** Serum estradiol and progesterone in pregnant group and nonpregnant group ($\bar{x}\pm s$).

Group	Estradiol (pg/mL)	Progesterone (ng/mL)
Pr group (*n* = 30)		
First trimester	24609.67 ± 18176.32^d^	76.10 ± 30.59^d^
Second trimester	62142.00 ± 23346.25^c^	123.84 ± 37.36^c^
Third trimester	81307.00 ± 32481.23^b^	210.16 ± 92.27^b^
N-Pr group (*n* = 20)		
First month	1438 ± 413.4018^a^	18.85 ± 9.95^a^
Second month	1470 ± 369.3237^a^	20.47 ± 12.60^a^
*F*	73.87	64.23
*P*	<0.0001	<0.0001

Pr group: pregnancy group; N-Pr group: nonpregnancy group. Different letters represent significant difference.
